# *Lacticaseibacillus rhamnosus* R0011 secretome attenuates *Salmonella enterica* serovar Typhimurium secretome-induced intestinal epithelial cell monolayer damage and pro-inflammatory mediator production in intestinal epithelial cell and antigen-presenting cell co-cultures

**DOI:** 10.3389/fmicb.2022.980989

**Published:** 2022-09-28

**Authors:** Michael P. Jeffrey, Chad W. MacPherson, Thomas A. Tompkins, Julia M. Green-Johnson

**Affiliations:** ^1^Applied Bioscience Graduate Program and the Faculty of Science, Ontario Tech University, Oshawa, ON, Canada; ^2^NutraPharma Consulting Services, Inc., Montreal, QC, Canada; ^3^Lallemand Bio-Ingredients, Inc., Montreal, QC, Canada

**Keywords:** macrophage inhibitory factor (MIF), *Lacticaseibacillus rhamnosus* R0011 secretome, Transwell, *Salmonella enterica* serovar Typhimurium secretome, intestinal epithelial cell, cytokines, antigen-presenting cell (APC), monocyte

## Abstract

Certain lactic acid bacteria (LAB) are associated with immune modulatory activities including down-regulation of pro-inflammatory gene transcription and expression. While host antigen-presenting cells (APCs) and intestinal epithelial cells (IEC) can interact directly with both pathogenic and commensal bacteria through innate immune pattern recognition receptors, recent evidence indicates indirect communication through secreted molecules is an important inter-domain communication mechanism. This communication route may be especially important in the context of IEC and APC interactions which shape host immune responses within the gut environment. We have previously shown that the *Lacticaseibacillus rhamnosus* R0011 secretome (LrS) dampens pro-inflammatory gene transcription and mediator production from Tumor Necrosis Factor-α and *Salmonella enterica* serovar Typhimurium secretome (STS)-challenged HT-29 IECs through the induction of negative regulators of innate immunity. However, many questions remain about interactions mediated through these bacterial-derived soluble components and the resulting host immune outcomes in the context of IEC and APC interactions. In the present study, we examined the ability of the LrS to down-regulate pro-inflammatory gene transcription and cytokine production from STS-challenged T84 human IEC and THP-1 human monocyte co-cultures. Cytokine and chemokine profiling revealed that apically delivered LrS induces apical secretion of macrophage inhibitory factor (MIF) and down-regulates STS-induced pro-inflammatory mediator secretion into the apical and basolateral chambers of the T84/THP-1 co-culture. Transcriptional profiling confirmed these results, as the LrS attenuated STS challenge-induced *CXCL8* and *NF*κ*B1* expression in T84 IECs and THP-1 APCs. Interestingly, the LrS also reversed STS-induced damage to monolayer transepithelial resistance (TER) and permeability, results which were confirmed by *ZO-1* gene expression and immunofluorescence visualization of ZO-1 expression in T84 IEC monolayers. The addition of a MIF-neutralizing antibody abrogated the ability of the LrS to reverse STS-induced damage to T84 IEC monolayer integrity, suggesting a novel role for MIF in maintaining IEC barrier function and integrity in response to soluble components derived from LAB. The results presented here provide mechanistic evidence for indirect communication mechanisms used by LAB to modulate immune responses to pathogen challenge, using *in vitro* approaches which allow for IEC and APC cell communication in a context which more closely mimics that which occurs *in vivo*.

## Introduction

Interactions between IECs and APCs help to shape the immune outcomes of bidirectional host-microbe communication at the gut mucosal interface. While IECs provide a physical barrier at the external interface of the intestinal lumen, they are also important mediators of host-microbe communication by integrating microbial-derived signals to the underlying APC populations (Peterson and Artis, 2014; Goto, 2019). As the primary sensors of microbial activity in the gut environment, IECs have the capacity to induce and influence antimicrobial and immunoregulatory activity of underlying APCs through cytokine and mediator production induced by signal transduction events following IEC PRR recognition of microbial components. Due to the constant barrage of microbial signals, IECs must tightly regulate their activation in order to maintain a state of homeostasis and reduce hyperresponsiveness to the normal gut microbiota (Burgueno and Abreu, 2020). For this reason, TLR expression is often limited to the basolateral side of polarized IECs, limiting the extent of interactions with microbial components found within the luminal contents of the intestine. For example, TLR5, which is responsible for recognizing bacterial flagella, is typically limited to the basolateral side of polarized IECs (Gewirtz et al., 2001). This enables IECs to respond to flagellated bacteria only if there is a breakdown of the epithelial barrier or translocation of the bacterium across the epithelial barrier, necessitating the need for a robust inflammatory response from the surrounding immune cell population to clear out the invading bacteria. The consequences of TLR recognition of microbial contents can also be dependent on whether the ligand is recognized on the apical or basolateral side of polarized IECs (Abreu, 2010). TLR9, which recognizes unmethylated CpG DNA sequences, is expressed on both the apical and basolateral side of polarized IECs. When TLR9 is activated on the apical surface of IECs, there is a muted response with the activation of genes involved in the regulation of NF-κB signaling, whereas basolateral recognition of CpG DNA induces the activation of classical NF-κB signaling (Lee et al., 2006), reinforcing the importance of examining immune outcomes in the context of polarized IECs and spatial compartmentalization of PRR-induced signaling within the gut.

IECs express tight junction proteins including the occludins, claudins, zonula occludens (ZO), and junctional adhesion molecules which work in concert to prevent the paracellular transport of intestinal luminal contents into the basolateral side of the epithelium (Gunzel and Yu, 2013; Suzuki, 2013). These proteins are tightly regulated and are key cellular players in the maintenance of normal barrier integrity and function (Karczewski and Groot, 2000; Harhaj and Antonetti, 2004; Marchiando et al., 2010). As such, perturbations in their activity can lead to the breakdown of the gut epithelial barrier resulting in the activation of dysregulated immune activity within the underlying APC population and the potential for dissemination of luminal contents and bacteria into systemic circulation. Certain pro-inflammatory cytokines, such as IFN-γ, can act to increase intestinal barrier permeability by reducing ZO-1 expression and localization (Scharl et al., 2009), while some gut-associated pathogens, such as *S. enterica* serovar Typhimurium, can also produce virulence factors which selectively disrupt ZO-1 and other tight junction proteins allowing for their translocation across the intestinal barrier (Tafazoli et al., 2003; Boyle et al., 2006). Commensal microorganisms and LAB have been shown to strengthen epithelial barrier integrity, a mechanism believed to be used by these bacteria to enhance their capacity for host colonization, and to antagonize the detrimental impacts of certain gut-associated pathogens on the gut epithelium (Ohland and Macnaughton, 2010; Madsen, 2012). Although most studies examining the impacts of gut-associated bacteria on gut epithelial barrier integrity have focused on direct interactions between live bacteria and IECs, some have suggested a role of microbial-derived metabolites and soluble components in this context. For example, *E. coli* Nissle 1917 conditioned media increased Caco-2 IEC monolayer integrity (Stetinova et al., 2010) and secreted peptides from *Bifidobacterium infantis* reversed TNF-α and IFN-γ-induced IEC barrier damage (Ewaschuk et al., 2008).

*In vitro* approaches to study the interactions between epithelial cells and APCs have relied on the use of Transwell cell culture inserts. These cell culture inserts allow for the examination of gut barrier integrity and function by measuring the transport of apically delivered ions and other macromolecules across a monolayer of IECs grown on a microporous membrane (Donato et al., 2011). To achieve this, IECs are cultured until a polarized monolayer is formed within the Transwell insert, creating distinct apical, and basolateral compartments *in vitro*. IEC barrier function and permeability can then be readily studied following cell challenge by measuring the transepithelial electrical resistance (TER) and the flux of a fluorescently labeled sugar of known molecular weight across the IEC monolayer (Harhaj and Antonetti, 2004). T84 human IECs are a widely used cell line for *in vitro* study of IECs, and do not easily differentiate into a heterogenous cell population with altered phenotypic characteristics following polarization into a confluent monolayer, making them an ideal tool for studying IEC barrier function and permeability in response to cell challenge (Dharmsathaphorn and Madara, 1990; Hillgren et al., 1995; Donato et al., 2011). To facilitate the study of IEC and APC interactions with bacteria, APCs can be cultured in the basolateral chamber and bacteria and their soluble components can be administered into the apical chamber following IEC monolayer formation to simulate interactions found within the gut-mucosal interface. This approach provides a useful *in vitro* system to study the dynamics of microbe-mediated immune communication in the context of IEC and APC interactions.

Several chemokines and cytokines are involved in intestinal epithelial barrier disruption by intestinal pathogens, including IFN-γ, TNF-α, and CXCL8 (Hansen et al., 2013; Andrews et al., 2018; Paradis et al., 2021). Certain cytokines also play key roles in intestinal epithelial barrier maintenance and repair, including MIF (Vujicic et al., 2018, 2020), and we have previously found that the LrS induces MIF production by IECs (Jeffrey et al., 2020b). In addition, the innate immune regulators ATF3 and DUSP1/MKP-1 are involved in intestinal epithelial barrier regeneration and maintenance, the latter through regulation of the p38MAPK signaling pathway (Chang et al., 2015; Talavera et al., 2015; Glal et al., 2018; Sheng et al., 2020) while *NF*κ*B1* expression is implicated in intestinal barrier disruption (Al-Sadi et al., 2010; McDaniel et al., 2016). We have previously shown that the LrS has the capacity to modulate immune outcomes in both human IECs and APCs with transcriptional and cytokine/chemokine profiling revealing context-dependent and cell-type specific immunomodulatory activity of the LrS, including induction of negative regulators of innate immunity *ATF3* and *DUSP1* (Jeffrey et al., 2020a,b). However, many questions remain about the LrS in regulating host immune outcomes in the context of IEC and APC interactions. To date, most studies examining the effects of LAB and their secreted products have focused on host immune responses using a single cell type (IEC or APC) *in vitro* and therefore do not necessarily reflect the impact these bacteria may have on interactions between these key cell types involved in innate immunity. Recent evidence also suggests that the effects of LAB and their secreted factors can be very different when tested in co-cultures of human IECs and APCs (Bermudez-Brito et al., 2015). As such, the aim of this study was to examine the immunomodulatory impacts of the LrS using co-cultures of human IECs and APC, focusing on cytokines and chemokines involved in gut epithelial barrier disruption and maintenance, markers of gut barrier integrity, and on expression of genes involved in regulating innate immune activity and barrier integrity at the intestinal barrier level. Analysis of the effects of the LrS in Transwell systems using co-cultures of human IECs and APCs provides an approach to further investigate the potential role of microbial secretomes in modulating host immune activity at the gut mucosal interface.

## Materials and methods

### Bacterial culture

Lyophilized *Lactobacillus rhamnosus* R0011 was obtained from RIMAP (Montreal, Quebec, Canada). The LrS was prepared as previously described (Jeffrey et al., 2018). Briefly, bacteria were grown in deMan, Rogosa and Sharpe (MRS) medium (Difco, Canada) at 37°C for 17 h in a shaking incubator and then diluted in non-supplemented RPMI-1,640 medium and allowed to further propagate for an additional 23 h under the same conditions. Both the bacterial culture and controls were centrifuged at 3,000 × *g* for 20 min and filtered through a 0.22 μm filter (Progene, Canada) to remove any bacteria. The filtered supernatant samples were also subjected to size fractionation using < 10 kDa Amicon Ultra—15 centrifugal filter (EMD Millipore, MA, USA).

For preparation of the STS, bacteria were propagated overnight in tryptone soya broth (Oxoid) in a shaking incubator at 37°C. Overnight cultures were centrifuged at 3,000 × g for 20 min at 4°C and filtered through a 0.22 μm filter and the secretome was stored at −80°C.

### Cell culture and challenge assay and RNA extraction

The T84 human colorectal carcinoma cell line was obtained from the American Type Culture Collection (ATCC, #CCL-248) and was maintained in DMEM/F-12 medium supplemented with 10% bovine calf serum and 0.05 mg/mL gentamicin (Sigma-Aldrich, MO, USA) and were grown in 75 cm^2^ tissue culture flasks (Greiner-Bio-One, NC, USA) at 37°C, 5% CO_2_ in a humidified incubator (Thermo Fisher Scientific, MA, USA) as described previously (Jeffrey et al., 2018). T84s IECs were enumerated and viability determined using Trypan Blue following sub-culturing. Cells were then resuspended in complete culture medium (DMEM/F-12 medium supplemented with 10% bovine calf serum and 0.05 mg/mL gentamicin) and 1.0 × 10^6^ cells were seeded into 12-well Millicell hanging cell culture inserts with a pore size of 0.4 μM (EMD Millipore, MA, USA). To obtain polarized confluent monolayers, seeded T84 IECs were incubated at 37°C, 5% CO_2_ in a humidified incubator for 7 days, or until a minimum TER of 1,000 Ωcm^2^ as described previously (Sherman et al., 2005). T84 IEC cell culture medium was aspirated and replaced with fresh non-supplemented (no calf serum) DMEM/F12 medium containing the LrS (30%v/v), the STS (1% v/v), or a combination of these secretome challenges in the apical chamber of the hanging cell culture and THP-1 human monocyte cells were then added to the basolateral chamber at a concentration of 1 × 10^6^ cells/mL for 24 h. These secretome concentrations have been previously shown to have immunomodulatory activity without negative impacts to cellular viability (Jeffrey et al., 2020b). For some challenges, cells were also cultured with an antibody specific for human MIF (AF-289-PB) (0.05 μg/mL) (R&D Systems) or with goat IgG isotype controls (AB-108-C) (0.05 μg/mL) (R&D Systems). This anti-MIF antibody has been used successfully to block the activity of MIF produced by IECs (Man et al., 2008). Following challenge, supernatants were collected from both the apical and basolateral sides of the Transwell inserts. TER measurements were done in triplicate using the Millicell ERS-2 Voltohmmeter (Millipore Sigma, MA, USA) to determine changes in epithelial monolayer integrity compared to controls and initial readings. Total RNA was also harvested after exposure to the various challenges from both T84 IECs and THP-1 human monocytes using the phenol-based TRIzol method of RNA extraction (Chomczynski, 1993) following manufacturer’s protocols (Thermo Fisher Scientific, MA, USA). Briefly, 2 mL of TRIzol reagent was added to each culture flask to lyse the IEC. Cell culture homogenates were added to Phase Lock Gel-Heavy tubes for phase separation of total RNA. Total extracted RNA was then purified using the RNeasy Plus Mini Kit (Qiagen, Hildon, Germany). The purity and quality of RNA was determined using a BioDrop Duo Spectrophotometer.

### Paracellular flux measurement

To determine the impact of the LrS or the STS on the permeability of T84 IEC monolayers, the flux of FITC-dextran across the epithelial monolayer was determined. Following cell challenge, T84 monolayers were washed with Hank’s Balanced Salt Solution (HBSS) (Millipore Sigma). Following washing, HBSS was added into the apical and basolateral chambers of the hanging cell culture insert and allowed to equilibrate for 30 min in a 37°C, 5% CO^2^ humidified incubator. The HBSS in the apical chamber was then replaced with HBSS containing 1 mg/mL of 4 kDa FITC-dextran (Millipore Sigma) and allowed to incubate for 1 h. Following incubation, a sample from the basolateral chamber was taken and placed into a black 96-well plate and fluorescence was quantified using a Synergy HT Microplate Reader (BioTek Instruments) set to 485/20 excitation and a 535/20 emission filter pair and a PMT sensitivity of 55. A FITC-dextran standard was used to quantify the concentration of FITC-dextran crossing the epithelial monolayer. This was repeated every hour for a total of 6 h and the transepithelial flux was determined by taking the average concentration of FITC-dextran and dividing by the surface area of the hanging cell culture insert; this was expressed as nM/cm^2^/h (Sanders et al., 1995).

### Comparative RT-qPCR

DNase-treated RNA (1 μg) from controls and each challenge were reverse transcribed with Superscript IV following manufacturer’s protocols as previously described (Macpherson et al., 2014). Reverse-transcribed cDNA was diluted 1:4 prior to amplification and 2.5 μL of diluted cDNA was used with SsoAdvanced Universal SYBR Green Supermix (Bio-Rad, CA, USA) in RT-qPCR. Gene-specific primers for targets identified as being potentially important in LrS-mediated impacts on IEC and THP-1 monocyte activity as described previously were used (Table 1; Jeffrey et al., 2020a,b). An initial incubation of 5 min at 95°C was performed, followed by 40 cycles consisting of template denaturation (15 s at 95°C) and one-step annealing and elongation (30 s at 60°C), with a Bio-Rad CFX Connect instrument (Bio-Rad, CA, USA). Four biological replicates were analyzed for each gene tested, and fold change expression levels were normalized to the expression levels of two reference genes for T84 IECs (*RPLPO* and *B2M*) and THP-1 monocytes (*RPL37A* and *ACTB*) and negative controls using Bio-Rad CFX Manager 3.1 software.

**TABLE 1:** List of primers used for relative RT-qPCR for determination of gene expression profiles in T84 IECs and THP-1 monocyte co-cultures.

Gene	GenBank accession number	Amplicon length (bp)	Primer sequence (5′–3′)	Source
*B2M*	NM_004048	150	F: GTGCTCGCGCTACTCTCTC	Dydensborg et al., 2006
			R: GTCAACTTCAATGTCGGAT	
*RPLPO*	NM_001002	142	F: GCAATGTTGCCAGTGTCTG	Dydensborg et al., 2006
			R: GCCTTGACCTTTTCAGCAA	
*TRIB3*	NM_021158.4	184	F: TGGTACCCAGCTCCTCTACG	Jousse et al., 2007
			R: GACAAAGCGACACAGCTTGA	
*ATF3*	NM_001206484.3	71	F: AAGAACGAGAAGCAGCATTTGAT	Bottone et al., 2005
			R: TTCTGAGCCCGGACAATACAC	
*DUSP1*	NM_004417	80	F: GGCCCCGAGAACAGACAAA	Locati et al., 2002
			R: GTGCCCACTTCCATGACCAT	
*NFêB1*	NM_003998.3	130	F: GCAGCACTACTTCTTGACCACC	Macpherson et al., 2014
			R: TCTGCTCCTGAGCATTGACGTC	
*CLDN1*	NM_021101.5	81	F: CCTATGACCCCAGTCAATGC	Tisza et al., 2016
			R: TCCCAGAAGGCAGAGAGAAG	
*CLDN3*	NM_001306.4	161	F: GTCCGTCCGTCCGTCCG	Rangel et al., 2003
			R: GCCCAGCACGGCCAGC	
*ZO-1*	NM_001330239.4	102	F: AAGTCACACTGGTGAAATCC	Boeckx et al., 2015
			R: CTCTTGCTGCCAAACTATCT	
*IL1R2*	NM_004633.4	101	F: TGTGCTGGCCCCACTTTC	Mar et al., 2015
			R: GCACAGTCAGACCATCTGCTTT	
*IDO1*	NM_002164.6	119	F: GCCTGATCTCATAGAGTCTGGC	Zhou et al., 2019
			R: TGCATCCCAGAACTAGACGTGC	
*CD36*	NM_001001548.3	118	F: CAGGTCAACCTATTGGTCAAGCC	Chen et al., 2020
			R: GCCTTCTCATCACCAATGGTCC	
*TLR1*	NM_003263.4	105	F: CAGTGTCTGGTACACGCATGGT	Lv et al., 2018
			R: TTTCAAAAACCGTGTCTGTTAAGAGA	
*DC-SIGN*	NM_001144893.2	136	F: TCAAGCAGTATTGGAACAGAGGA	Chanput et al., 2013
			R: CAGGAGGCTGCGGACTTTTT	
*CD206*	NM_002438.4	97	F: CAGCGCTTGTGATCTTCATT	Chanput et al., 2013
			R: TACCCCTGCTCCTGGTTTTT	
*ZFP36L1*	NM_001244698.2	116	F: ATGACCACCACCCTCGTGT	Chen et al., 2015
			R: TTTCTGTCCAGCAGGCAACC	
*ACTB*	NM_001101.5	150	F: ATTGCCGACAGGATGCAGAA	Maess et al., 2010
			R: GCTGATCCACATCTGCTGGAA	
*RPL37A*	NM_000998.5	94	F: ATTGAAATCAGCCAGCACGC	Maess et al., 2010
			R: AGGAACCACAGTGCCAGATCC	

### Morphological characterization

Changes in T84 IEC monolayer integrity following challenge with the LrS or the STS was visualized by staining ZO-1 with an anti-ZO-1 monoclonal antibody (ZO1-1A12) conjugated with Alexa Fluor 488 (Thermo Fisher Scientific), following manufacturing protocols. Briefly, following conditioning, cells were fixed with 3.75% formaldehyde, permeabilized with 0.5% Triton X-100, and stained with anti-ZO for 2 h at room temperature. Cells were counter-stained and mounted using ProLong™ Diamond Antifade Mountant with DAPI (Thermo Fisher Scientific Cat #: P36966).

### Cytokine/chemokine/inflammatory marker analysis

Cell culture supernatants from both the apical and basolateral chambers of the hanging cell culture inserts were collected following 24 h of challenge in order to allow sufficient time for the production of key inflammatory cytokines and chemokines and to determine directionality of cytokine release. Cytokine and chemokine profiling was performed using the Bio-Plex Pro™ 40-Plex Human Chemokine Panel (Bio-Rad #171ak99mr2) and the Bio-Plex Pro™ Human Inflammation Panel 1, 37-Plex (Bio-Rad #171AL001M). All 40 chemokines (CCL21, BCA-1/CXCL13, CTACK/CCL27, ENA-78/CXCL5, Eotaxin/CCL11, Eotaxin-2/CCL24, Eotaxin-3/CCL26, Fractalkine/CX3CL1, GCP-2/CXCL6, GM-CSF, Gro-α/CXCL1, Gro-β/CXCL2, I-309/CCL1, IFN-υ, IL-1β, IL-2, IL-4, IL-6, IL-8/CXCL8, IL-10, IL-16, IP-10/CXCL10, I-TAC/CXCL11, MCP-1/CCL2, MCP-2/CCL8, MCP-3/CCL7, MCP-4/CCL13, MDC/CCL22, MIF, MIG/CXCL9, MIP-1α/CCL3, MIP-1δ/CCL15, MIP-3α/CCL20, MIP-3β/CCL19, MPIF-1/CCL23, SCYB16/CXCL16, SDF-1α + β/CXCL12, TARC/CCL17, TECK/CCL25, TNF-α) or 37 cytokines [APRIL/TNFSF13, BAFF/TNFSF13B, sCD30/TNFRSF8, sCD163, Chitinase-3-like 1, gp130/sIL-6Rβ, IFN-α2, IFN-β, IFN-γ, IL-2, sIL-6Rα, IL-8, IL-10, IL-11, IL-12 (p40), IL-12 (p70), IL-19, IL-20, IL-22, IL-26, IL-27 (p28), IL-28A/IFN-λ2, IL-29/IFN-λ1, IL-32, IL-34, IL-35, LIGHT/TNFSF14, MMP-1, MMP-2, MMP-3, Osteocalcin, Osteopontin, Pentraxin-3, sTNF-R1, sTNF-R2, TSLP, TWEAK/TNFSF12] were multiplexed on the same 96-well plate. Chemokine/cytokine standards were serially diluted and chemokine profiling from all cell challenges was done following manufacturer’s instructions (Bio-Rad, CA, USA) with 4 biological replicates. Quality controls were also included to ensure the validity of the concentrations that were obtained. The Bio-Plex Manager™ software was used to determine the concentration of the analytes within each sample using the generated standard curves and concentration was expressed in pg/mL (concentration in range). Statistical analysis was done using GraphPad Prism’s (Version 8) one-way analysis of variance (ANOVA) and Tukey’s multiple comparison test when the ANOVA indicated significant differences were present. All data are shown as the mean pg/mL ± standard error of the mean (SEM). Z-scores were determined and visualized using R version 4.0.0 and package Bioconductor to determine the overall impact of each challenge on cytokine and chemokine production from T84 IEC and THP-1 monocyte co-cultures.

### Flow cytometry

Differential cell-surface marker expression of CD74 on T84 IECs challenged with the LrS, STS, LrS, and STS or medium controls was determined using a BD Accuri C6 flow cytometer. Following challenge, 1 × 10^6^ cells were resuspended in D-phosphate buffered saline (D-PBS) and cells were stained with the viability dye 7-AAD (Tonbo, 13–6,993) for 10 min on ice while protected from light. Cells were washed with 1 mL of cell staining buffer (CSB) (4% calf serum, 5 mM EDTA in D-PBS) and centrifuged for 5 min at 400 × *g* (4°C). Immediately following viability staining, non-specific Fc-mediated interactions were blocked by the addition of 100 μL of blocking buffer [10% calf serum (heat inactivated) in D-PBS] to the cell suspension for 10 min. Anti-human CD74 (BioLegend, clone LN2) was added to the cell suspension followed by incubation on ice and protected from light for 30 min. Cells were washed with CSB as described above, resuspended in 100 μL of fixation buffer (4% paraformaldehyde-PBS) and incubated for 30 min at room temperature protected from light. Data acquisition was done using the BD Accuri Plus flow cytometer and corresponding software package. 30,000 viable cells (as determined by incorporation of 7-AAD viability dye [Tonbo 13–6,993)] were acquired for each experiment and subsequent analysis was done using FlowJo v. 10.

## Results

### The *Lacticaseibacillus rhamnosus* R0011 secretome attenuates serovar Typhimurium secretome-induced pro-inflammatory cytokine and chemokine production from T84 intestinal epithelial cells and THP-1 APC co-cultures

**FIGURE 1:**
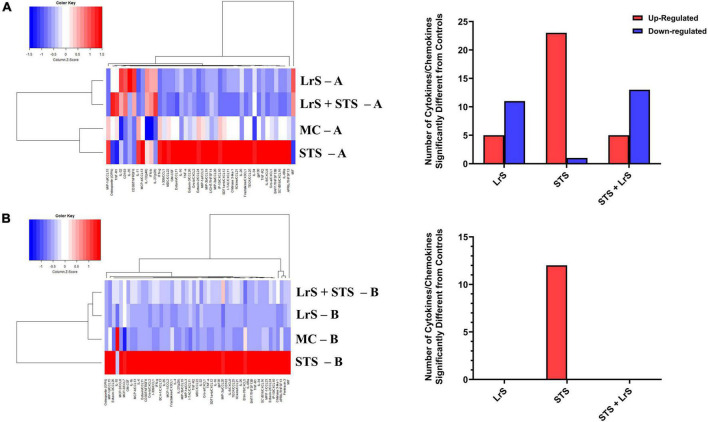
Two-dimensional hierarchical clustering analysis of cytokine and chemokine production profiles from the **(A)** apical (A) and **(B)** basolateral (B) chambers of T84 IEC/THP-1 human monocytes in response to apically delivered LrS, STS, a combination of the LrS and the STS, or medium controls for 24 h. Data shown is the Z-score statistic for each cytokine and chemokine measured (*n* = 4).

**FIGURE 2:**
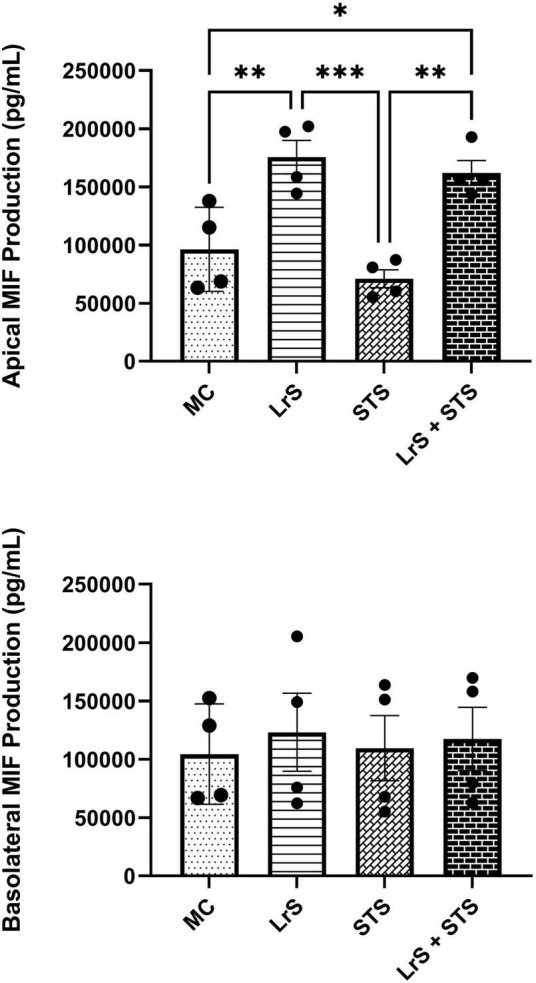
Apical and basolateral production of MIF by T84 IEC and THP-1 monocyte co- cultures following challenge with the LrS, STS, or a combination of the secretomes for 24 h. Data shown is the mean cytokine/chemokine production (pg/mL) ± SEM (*n* = 4). Significance is indicated as ^∗^*p* < 0.05, ^∗∗^*p* < 0.01, ^∗∗∗^*p* < 0.001 as determined by one-way ANOVA and Tukey’s *post-hoc* test.

Cytokine and chemokine profiling of IEC and APC co-cultures was done to determine whether apically delivered LrS and STS could alter functional immune outcomes in co-cultures of T84 IEC monolayers and THP-1 human monocytes. STS-challenge resulted in increased production of CCL1, CCL15, CCL20, CCL21, CCL24, CCL26, CX3CL1, CXCL1, CXCL2, CXCL8, CXCL10, CXCL11, CXCL16, IFN-γ, TNF-α, TNFSF13, TNFSF13B, C3L1, and gp130/sIL-6Rβ into the apical chamber of the co-culture system when compared to controls (*n* = 4; *p* < 0.05) (Figure 1 and Supplementary Figures 1A, B). This correlated with increased production of CCL15, CCL21, CCL23, CCL27, CX3CL1, GM-CSF, CXCL1, CXCL8, IFNγ, IL-6RA, IL-16, and C3L1 found within the basolateral chamber of the co-culture system when compared to controls, suggesting that the STS has the capacity to influence immune effector function of cells found beneath an IEC monolayer (*n* = 4; *p* < 0.05) (Figure 1 and Supplementary Figure 2). In contrast, challenge with the LrS only resulted in increased apical production of CD163, IL-32, IL-34, MIF, and TNFR2, with no significant impact on cytokines and chemokines found within the basolateral chamber (*n* = 4; *p* < 0.05) (Figures 1, 2 and Supplementary Figure 3). Challenge of IEC-APC co-cultures with a combination of the STS and LrS resulted in attenuation of all STS-induced pro-inflammatory mediator production to constitutive levels in both the apical and basolateral chambers of the co-culture system, indicating that apically delivered LrS can attenuate STS-induced inflammatory mediator production in co-cultures of IECs and APCs (*p* < 0.05; Figure 1).

### The *Lacticaseibacillus rhamnosus* R0011 secretome attenuates serovar Typhimurium secretome-induced pro-inflammatory gene expression in T84 intestinal epithelial cells and THP-1 monocytes

**FIGURE 3:**
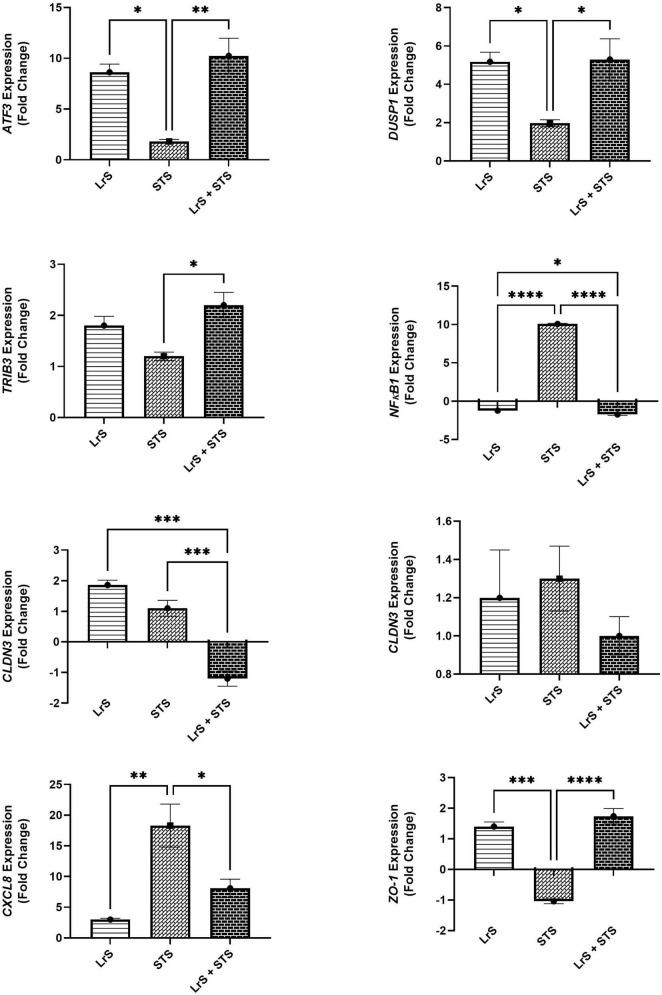
Gene expression profiles of T84 IECs in T84 IEC/THP-1 monocyte co-cultures challenged with the LrS, STS, or a combination of the challenges. Data shown is the mean fold- change relative to untreated controls ± SEM (*n* = 4). Significance is indicated as ^∗^*p* < 0.05, ^∗∗^*p* < 0.01, ^∗∗∗^*p* < 0.001, ^∗∗∗∗^*p* < 0.0001 as determined by one-way ANOVA and Tukey’s *post-hoc* test.

**FIGURE 4:**
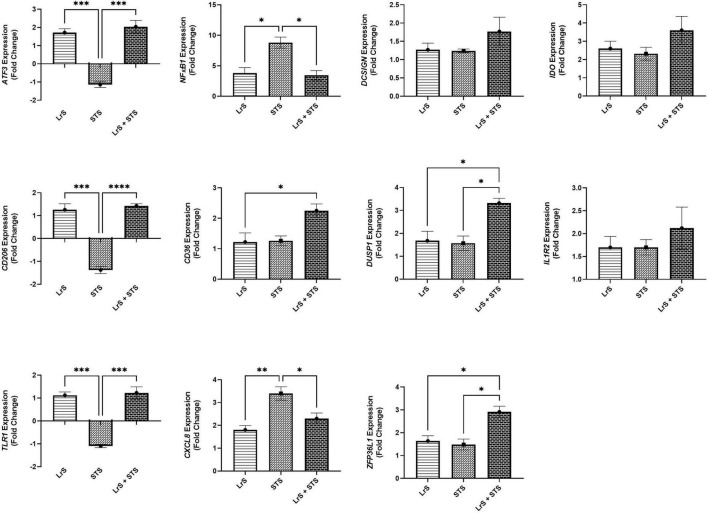
Gene expression profiles of THP-1 monocytes in T84 IEC/THP-1 monocyte co-cultures challenged with the LrS, STS, or a combination of the challenges. Data shown is the mean fold-change relative to untreated controls ± SEM (*n* = 4). Significance is indicated as ^∗^*p* < 0.05, ^∗∗^*p* < 0.01, ^∗∗∗^*p* < 0.001, ^∗∗∗∗^*p* < 0.0001 as determined by one-way ANOVA and Tukey’s *post-hoc* test.

Analysis of changes in gene-expression profiles in co-cultures of T84 IECs and THP-1 monocytes in response to challenge with the LrS or the STS was carried out to interrogate potential mechanism(s) of action behind the immunomodulatory activity of the LrS observed in individual cell populations. Consistent with the results obtained from the cytokine/chemokine profiling, STS-challenge of T84 IECs resulted in increased transcription of *CXCL8* (*n* = 3; *p* < 0.05) (Figure 3), an effect also seen in the underlying THP-1 monocyte cell population (Figure 4). STS-challenge also up-regulated the transcription of *NF*κ*B1*, a key transcription factor in the NF-κB signaling complex, in T84 IECs (*n* = 3; *p* < 0.05) and THP-1 monocytes (*n* = 3; *p* < 0.05) (Figures 3, 4). Interestingly, STS challenge down-regulated the expression of *ZO-1*, a gene encoding a tight-junction associated protein, indicating that the STS may be impacting tight-junctions within T84 IEC monolayers (*n* = 3; *p* < 0.05) (Figure 3). Concurrent challenge of co-cultures of T84 IECs and THP-1 monocytes with the LrS and the STS resulted in the attenuation of STS-induced transcription of *CXCL8* and *NF*κ*B1* in both T84 IECs and THP-1 monocytes, coupled with increased transcription of *ZO-1* and of *ATF3* and *DUSP1*, negative regulators of innate immunity, in T84 IECs (*n* = 3; *p* < 0.05) (Figures 3, 4). Moreover, concurrent challenge of co-cultures of T84 IECs and THP-1 monocytes with the LrS and the STS up-regulated the expression of *ZFP36L1*, *CD36*, and *ATF3* in the underlying THP-1 monocyte cell population when compared to STS- or LrS-challenge alone (*n* = 3; *p* < 0.05) (Figure 4).

### The *Lacticaseibacillus rhamnosus* R0011 secretome reverses serovar Typhimurium secretome-induced damage to T84 intestinal epithelial cell monolayer integrity via increased macrophage inhibitory facto*r* production

**FIGURE 5:**
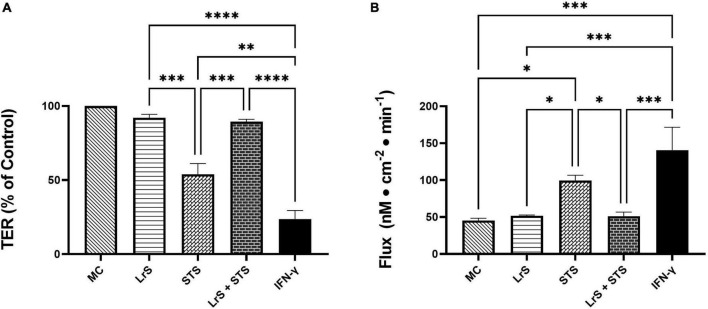
The LrS attenuates STS-induced damage to T84 IEC monolayer integrity and permeability. **(A)** Transepithelial resistance (TER) was measured following challenge of T84 IECs with the LrS, STS, combined secretome challenges, or IFN-γ (100 ng/mL) for 24 h (*n* = 4). **(B)** T84 IEC monolayer permeability was determined by measuring the amount of flux of FITC-dextran following challenge (*n* = 4). Significance is indicated as ^∗^*p* < 0.05, ^∗∗^*p* < 0.01, ^∗∗∗^*p* < 0.001, ^∗∗∗∗^*p* < 0.0001 as determined by one-way ANOVA and Tukey’s *post-hoc* test.

**FIGURE 6:**
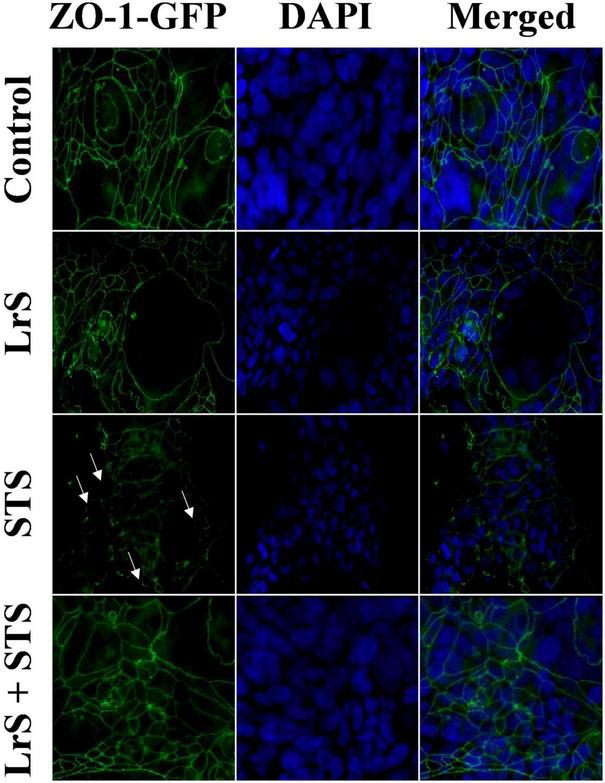
The LrS attenuates STS-induced damage to T84 IEC monolayer and integrity by antagonizing STS damage to ZO-1. Confluent T84 IECs were stained with anti-ZO-1-GFP and counterstained with DAPI following 24 h of challenge with the LrS, STS, LrS + STS, or medium controls and visualized at 100X under oil immersion.

**FIGURE 7:**
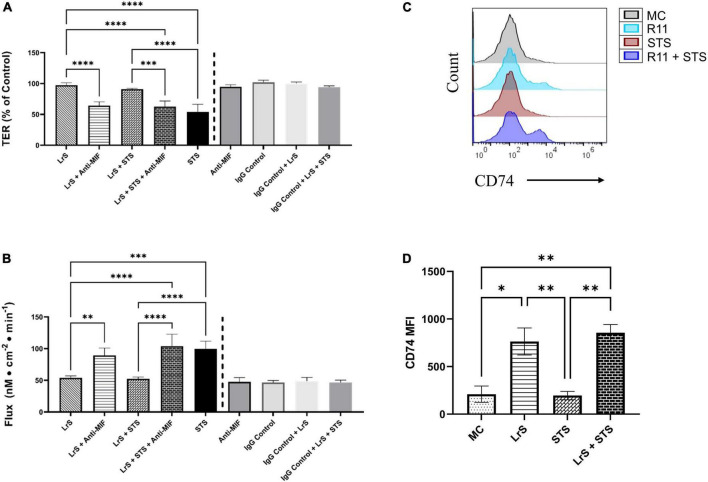
The impact of the LrS on T84 IEC barrier function and integrity is abrogated by the addition of a MIF blocking antibody. **(A)** Transepithelial resistance (TER) was measured following challenge of T84 IECs with the LrS, STS, or a combination of the challenges with an anti-MIF antibody or isotype controls (0.5 μg/mL) for 24 h (*n* = 3). **(B)** T84 IEC monolayer permeability was determined by measuring the amount of flux of FITC-dextran following challenge (*n* = 3). Significance is indicated as ^∗^*p* < 0.05, ^∗∗^*p* < 0.01, ^∗∗∗^*p* < 0.001 as determined by one-way ANOVA and Tukey’s *post-hoc* test. **(C)** Histogram analysis was used to determine the relative number of T84 IECs expressing CD74 following challenge with the LrS (blue), STS (red), LrS and STS (purple), or untreated (black) controls. **(D)** Median fluorescence intensity (MFI) of T84 IECs expressing CD74 was used to confirm the results of the histogram analysis. Data shown is the mean of the MFI ± SEM (*n* = 3). Significance is indicated as ^∗^*p* < 0.05, ^∗∗^*p* < 0.01, ^∗∗∗^*p* < 0.001, ^∗∗∗∗^*p* < 0.0001 as determined by one-way ANOVA and Tukey’s *post-hoc* test.

To further interrogate potential underlying mechanism(s) of action behind the observed bioactivity of the LrS in the context of STS challenge within a co-culture system, the impact of the LrS and STS on IEC barrier integrity and permeability was determined. The LrS had no significant impact on TER measurements of T84 IEC monolayers, with no significant changes to the paracellular flux of FITC-dextran when compared to controls (*n* = 4; *p* > 0.05) (Figure 5), indicating no detrimental impacts on IEC monolayer integrity and permeability. In contrast, T84 IECs challenged with the STS had a significantly lower TER measurement than cells incubated with the LrS (*n* = 4; *p* < 0.05) (Figure 5A), confirming the results obtained by RT-qPCR analysis which indicated that the STS reduced the expression of tight junction proteins. This correlated with significantly higher amounts of paracellular flux of FITC-dextran when compared to controls and cells challenged with the LrS (*n* = 4; *p* < 0.05) (Figure 5), indicating overall deleterious impacts of the STS on IEC monolayer function and integrity. Co-challenge of STS-challenged T84 monolayers with the LrS resulted in no significant alterations in TER or paracellular flux, suggesting that the LrS can antagonize the negative impacts of STS-challenge on IEC monolayer integrity (*n* = 4; *p* < 0.05) (Figure 5). Morphological changes in T84 monolayers confirmed these findings as T84 IECs challenged with the STS displayed broken tight junctions via reduced expression of ZO-1 (Figure 6). In contrast, T84 IECs challenged with the LrS or co-challenged with both the STS and LrS had intact tight junctions (Figure 6). The addition of a MIF neutralizing antibody abrogated the observed bioactivity of the LrS on STS-challenged T84 IEC barrier integrity and function (Figures 7A,B), suggesting that LrS-induced MIF production may play a protective role in maintaining IEC barrier integrity. Moreover, the cell-surface expression of CD74, one of the cellular receptors for MIF, was higher on T84 IECs challenged with the LrS or co-challenged with both the STS and LrS (Figures 7C,D).

## Discussion

Interactions between IECs and the underlying APC population act in concert to shape immune responses to apically sensed bacteria and their soluble mediators. While current evidence suggests that certain LAB and gut-associated pathogenic bacteria or their soluble mediators can influence host immune outcomes in single cell *in vitro* cell cultures, less is known about their impacts on IEC and APC co-cultures. Moreover, the impact of soluble mediators derived from both LAB and gut-associated pathogens on IEC monolayer integrity and subsequent activity on the underlying APC population remains unknown, with recent evidence suggesting that this is an important route of host-microbe immune communication within the GALT. Cytokine and chemokine profiling revealed that LrS challenge induced a muted response from T84 IEC and THP-1 monocyte co-cultures, with no significant changes in the production of cytokines and chemokines from THP-1 monocytes found within the basolateral chamber of the co-culture system. However, the LrS induced the production of IL-32 into the apical compartment of the co-culture system. Although IL-32 has been associated with the pathophysiology of inflammatory bowel disease (Khawar et al., 2016), evidence suggests that it may also play an immunoregulatory role in the progression of disease. For example, IL-32 can inhibit TNF-α-induced IL-8 production by inhibiting the translation of IL-8 mRNA into functional protein through an unknown mechanism (Imaeda et al., 2011). LrS challenge also induced the production of MIF by T84 IECs into the apical chamber, a result consistent with that seen previously in HT-29 IECs challenged with the LrS (Jeffrey et al., 2020b), indicating that LrS-induced MIF production is not limited to HT-29 IECs. MIF has pleiotropic roles in regulating immune outcomes in IECs. However, MIF also plays an integral role in initiating inflammatory responses in APCs through NLRP3 inflammasome activation and subsequent release of inflammatory mediators (Lang et al., 2018). Interestingly, LrS challenge did not increase MIF secretion into the basolateral chamber or induce MIF production from THP-1 monocytes, suggesting that LrS-induced MIF production is spatially compartmentalized into the apical chamber, limiting potential activation of the underlying THP-1 monocytes. Limiting the directionality of the release of certain cytokines and chemokines by IECs may serve as a means of muting bidirectional immune communication and subsequent activation of the underlying APC population within the GALT to certain challenges while still allowing for paracrine communication with adjacent IECs.

STS-challenge resulted in increased production of pro-inflammatory cytokines and chemokines found in the apical and basolateral chambers of the co-culture system. Indeed, previous evidence describes a route through which ST can invade the intestinal mucosa by infecting IECs and the underlying APC populations. However, induction of pro-inflammatory mediator production in co-cultures of T84 IECs and THP-1 monocytes in response to STS challenge represents a potential novel route of pathogenicity mediated through soluble mediators derived from gut-associated pathogens. While the mechanisms through which STS induced THP-1 cells to release proinflammatory cytokines and chemokines into the basolateral chambers remain to be determined, one possibility is that STS-mediated disruption of the T84 IEC barrier led to increased access of pro-inflammatory STS components to the THP-1 cells, directly resulting in heightened production of pro-inflammatory mediators by these APCs. Pro-inflammatory cytokine and chemokine production by THP-1 APCs may also be induced by pro-inflammatory cytokines released by STS-induced activation of T84 IECs, and it possible that both of these routes operate *in vivo* during Salmonella infection. Co-challenge with the LrS attenuated STS-induced pro-inflammatory mediator production from T84 IEC and THP-1 monocyte co-cultures, a result that is in keeping with the observed bioactivity of the LrS in STS-challenged HT-29 IECs (Jeffrey et al., 2020b). In contrast, *Lactobacillus paracasei* CNCM-4034 and its secretome was found to induce pro-inflammatory mediator production and enhance pro-inflammatory gene transcription in response to challenge with *Salmonella typhi* in Caco-2 IEC and APC co-cultures, illustrating differences in secretome-mediated effects of lactobacilli on immune activity (Bermudez-Brito et al., 2015).

Challenge with the LrS induced the expression of *ATF3* and *DUSP1*, but not *TRIB3* in T84 IECs. This is in contrast to previous transcriptional profiling of HT-29 IECs following LrS challenge, as induction of these negative regulators of innate immunity was only seen following co-challenge of HT-29 IECs with the LrS and TNF-α or the STS (Jeffrey et al., 2020b). THP-1 human monocytes were less responsive to LrS challenge than T84 IECs as there were no significant changes to gene expression profiles in the absence of pro-inflammatory challenge. Co-challenge of STS-challenged co-cultures with the LrS attenuated *NF-*κ*B1* as well as *CXCL8* gene expression in both T84 IECs and THP-1 monocytes, confirming the results obtained in the cytokine and chemokine profiling. As was seen in HT-29 IECs co-challenged with the LrS and the STS (Jeffrey et al., 2020b), there was also increased expression of *ATF3*, *DUSP1*, and *TRIB3* in STS-challenged T84 IECs following concurrent secretome challenge, suggesting conserved immunoregulatory activity of the LrS across different *in vitro* IEC models and in co-culture systems.

Interestingly, the STS-challenge also reduced the expression of *ZO-1*, a tight-junction protein integral to proper function of the IEC barrier, providing potential insight into STS-induced pro-inflammatory responses in THP-1 monocytes observed in the basolateral chamber of the co-culture system. Deterioration of IEC monolayer integrity through disruption of tight junction proteins such as ZO-1 is a hallmark of *S. enterica* serovar Typhimurium infection, resulting in enhanced translocation of the bacteria into the lamina propria (Boyle et al., 2006; Kohler et al., 2007). In keeping with these findings, the STS decreased T84 IEC TER and increased the flux of FITC-dextran across T84 IEC monolayers, indicating damage to IEC monolayer integrity and function. These results were further confirmed through visual inspection of ZO-1 expression following STS-challenge. Typically, damage to IEC monolayer integrity caused by *S. enterica* serovar Typhimurium infection is mediated by the direct delivery of virulence factors into host cells via a type III secretion system (Coburn et al., 2007a,b). However, the results presented here suggest that secretome components derived from *S. enterica* serovar Typhimurium grown under normal conditions can also disrupt normal IEC monolayer function. The LrS attenuated STS-induced damage to T84 IEC monolayer integrity and function. Although there have been other reports of LAB-mediated strengthening of IEC monolayer barrier integrity, the results presented here suggest a possible novel route through which soluble mediators derived from LAB can antagonize the activity of certain gut-associated pathogens.

Addition of a MIF-neutralizing antibody reversed the ability of the LrS to attenuate STS-induced damage to IEC epithelial monolayer integrity. Recent evidence has suggested that MIF is integral for IEC repair and regeneration as well as for normal barrier function of IECs, as MIF deficient mice have increased intestinal permeability due to impairment of tight junction proteins such as ZO-1 (Vujicic et al., 2018, 2020). Mechanistically, MIF binds to CD74 (Leng et al., 2003), typically following pro-inflammatory challenge or perturbations in normal IEC activity (Farr et al., 2020b). Interestingly, T84 IECs challenged with the LrS or co-challenged with both the STS and LrS had higher cell-surface receptor expression of CD74. The CD74-MIF signaling complex facilitates the activation of PI3K/AkT and ERK cell proliferation and survival cellular pathways (Lue et al., 2007; Farr et al., 2020a). However, the precise mechanism(s) involved in the induction of MIF production following LrS challenge remain unknown and warrant further study, especially in the context of polarized IECs which display vectoral secretion of certain cytokines and chemokines.

Co-culture in Transwell systems provides a useful strategy to interrogate the impact of microbial products on IEC and APC interactions, including LAB-mediated attenuation of pathogen-induced inflammatory responses. The results presented here provide mechanistic insight into the ability of the LrS to modulate immune responses to STS-challenge in a context that allows for IEC and APC cell communication. The LrS was able to reverse STS-induced damage to IEC monolayer integrity, an effect potentially mediated through LrS-induced MIF production by IECs. Further experimentation using additional timepoints and secretomes derived from different LAB may reveal species-specific and time-dependent consequences of prolonged exposure to soluble components from LAB in a context which more closely mimics that which occurs *in vivo.* These future studies would provide further potential insights into mechanisms of gut microbe-host immune communication at the gut-mucosal interface.

## Data availability statement

All relevant data are contained within the article. The original contributions presented in this study are included in the article/Supplementary material. Further inquiries can be directed to the corresponding author.

## Author contributions

MJ and CM carried out the experiments. MJ performed the statistical analysis and wrote the first draft of the manuscript. All authors contributed to manuscript revision, approved the submitted version, and contributed to the study design.

## Supplementary material

Supplementary Figure 1A Apical production of cytokines and chemokines which were significantly increased by STS treatment of T84 IEC and THP-1 monocyte co-cultures. Data shown is the mean cytokine/chemokine production (pg/mL) ± SEM (*n* = 4). Significance is indicated as ^∗^*p* < 0.05, ^∗∗^*p* < 0.01, ^∗∗∗^*p* < 0.001, ^∗∗∗∗^*p* < 0.0001 as determined by one-way ANOVA and Tukey’s *post-hoc* test.

Supplementary Figure 1B Apical production of cytokines and chemokines which were significantly increased by STS treatment of T84 IEC and THP-1 monocyte co-cultures. Data shown is the mean cytokine/chemokine production (pg/mL) ± SEM (*n* = 4). Significance is indicated as ^∗^*p* < 0.05, ^∗∗^*p* < 0.01, ^∗∗∗^*p* < 0.001, ^∗∗∗∗^*p* < 0.0001 as determined by one-way ANOVA and Tukey’s *post-hoc* test.

Supplementary Figure 2 Basolateral production of cytokines and chemokines which were significantly increased by STS treatment of T84 IEC and THP-1 monocyte co-cultures. Data shown is the mean cytokine/chemokine production (pg/mL) ± SEM (*n* = 4). Significance is indicated as ^∗^*p* < 0.05, ^∗∗^*p* < 0.01, ^∗∗∗^*p* < 0.001, ^∗∗∗∗^*p* < 0.0001 as determined by one-way ANOVA and Tukey’s *post-hoc* test.

Supplementary Figure 3 Apical production of cytokines and chemokines which were significantly increased by LrS treatment of T84 IEC and THP-1 monocyte co-cultures. Data shown is the mean cytokine/chemokine production (pg/mL) ± SEM (*n* = 4). Significance is indicated as ^∗^*p* < 0.05, ^∗∗^*p* < 0.01, ^∗∗∗^*p* < 0.001, ^∗∗∗∗^*p* < 0.0001 as determined by one-way ANOVA and Tukey’s *post-hoc* test.
