# Supplementary figures and images for: Lacticaseibacillus rhamnosus R0011 secretome attenuates Salmonella enterica serovar Typhimurium secretome-induced intestinal epithelial cell monolayer damage and pro-inflammatory mediator production in intestinal epithelial cell and antigen-presenting cell co-cultures
Source: Front Microbiol. 2022 Sep 28;13:980989. doi: 10.3389/fmicb.2022.980989 (PMC9554441; doi:10.3389/fmicb.2022.980989)

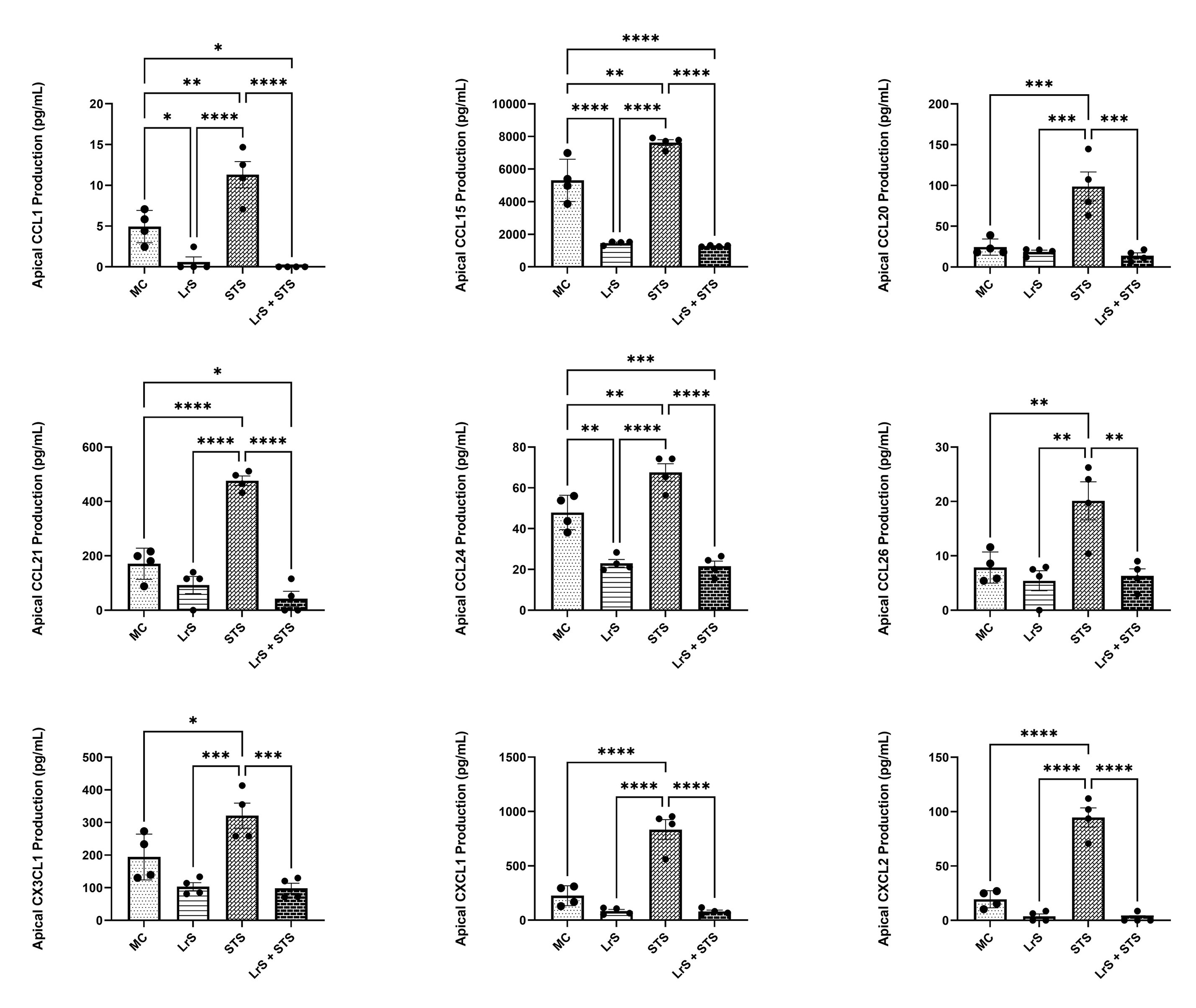

Supplement: Supplementary Figure 1A — Apical production of cytokines and chemokines which were significantly increased by STS treatment of T84 IEC and THP-1 monocyte co-cultures. Data shown is the mean cytokine/chemokine production (pg/mL) ± SEM (n = 4). Significance is indicated as ∗p < 0.05, ∗∗p < 0.01, ∗∗∗p < 0.001, ∗∗∗∗p < 0.0001 as determined by one-way ANOVA and Tukey’s post-hoc test. [file Image_1.JPEG]

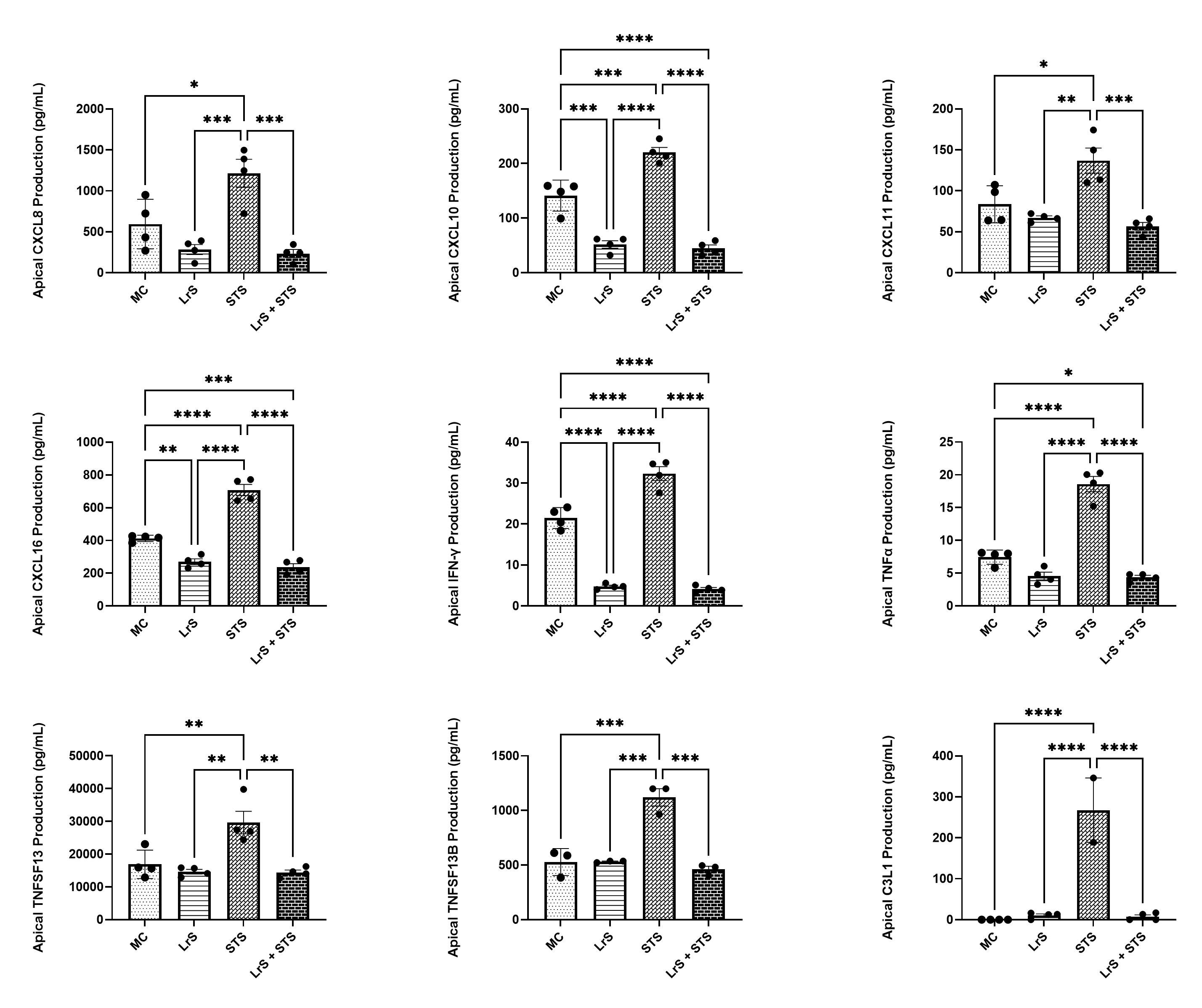

Supplement: Supplementary Figure 1B — Apical production of cytokines and chemokines which were significantly increased by STS treatment of T84 IEC and THP-1 monocyte co-cultures. Data shown is the mean cytokine/chemokine production (pg/mL) ± SEM (n = 4). Significance is indicated as ∗p < 0.05, ∗∗p < 0.01, ∗∗∗p < 0.001, ∗∗∗∗p < 0.0001 as determined by one-way ANOVA and Tukey’s post-hoc test. [file Image_2.JPEG]

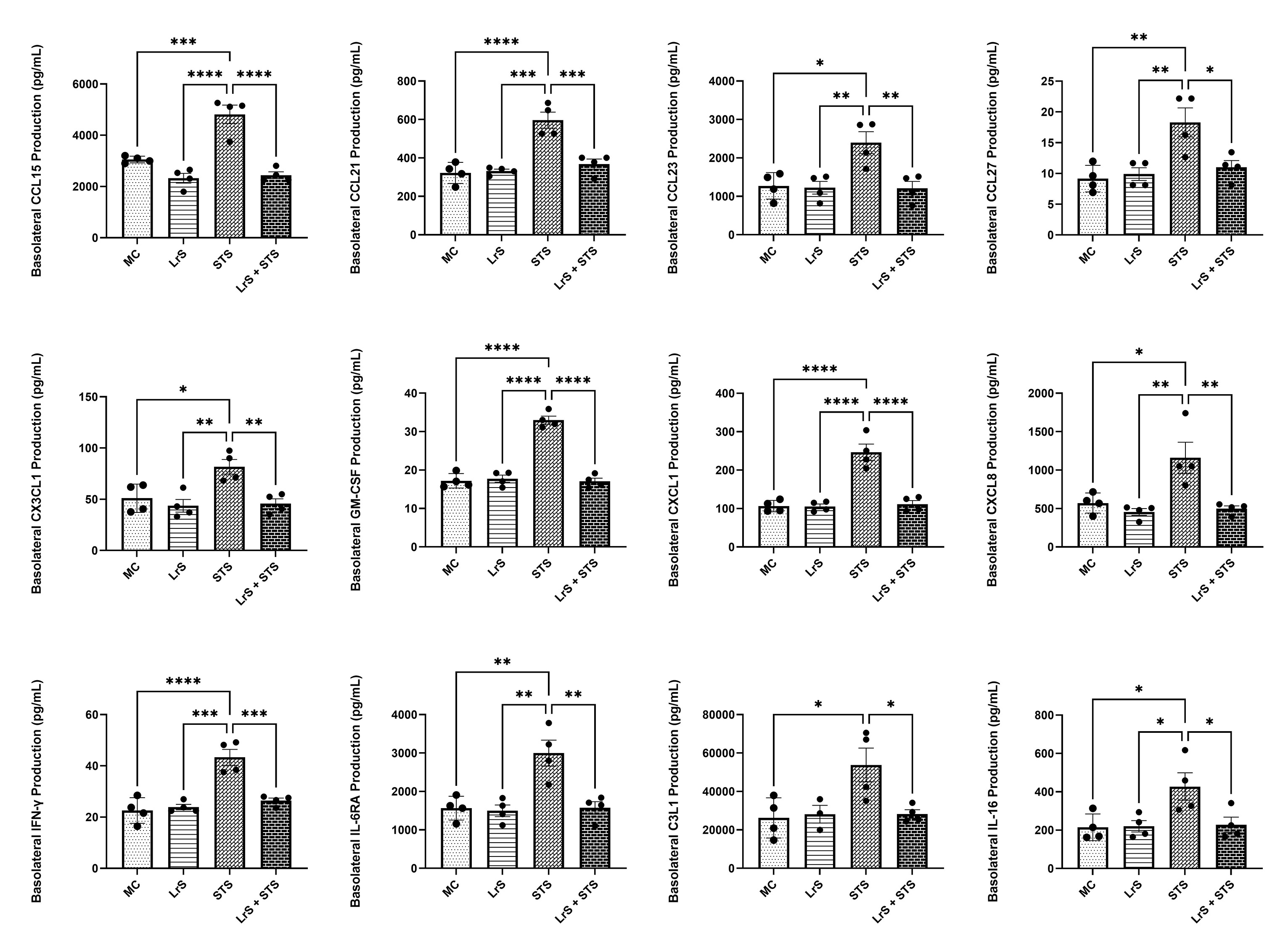

Supplement: Supplementary Figure 2 — Basolateral production of cytokines and chemokines which were significantly increased by STS treatment of T84 IEC and THP-1 monocyte co-cultures. Data shown is the mean cytokine/chemokine production (pg/mL) ± SEM (n = 4). Significance is indicated as ∗p < 0.05, ∗∗p < 0.01, ∗∗∗p < 0.001, ∗∗∗∗p < 0.0001 as determined by one-way ANOVA and Tukey’s post-hoc test. [file Image_3.JPEG]

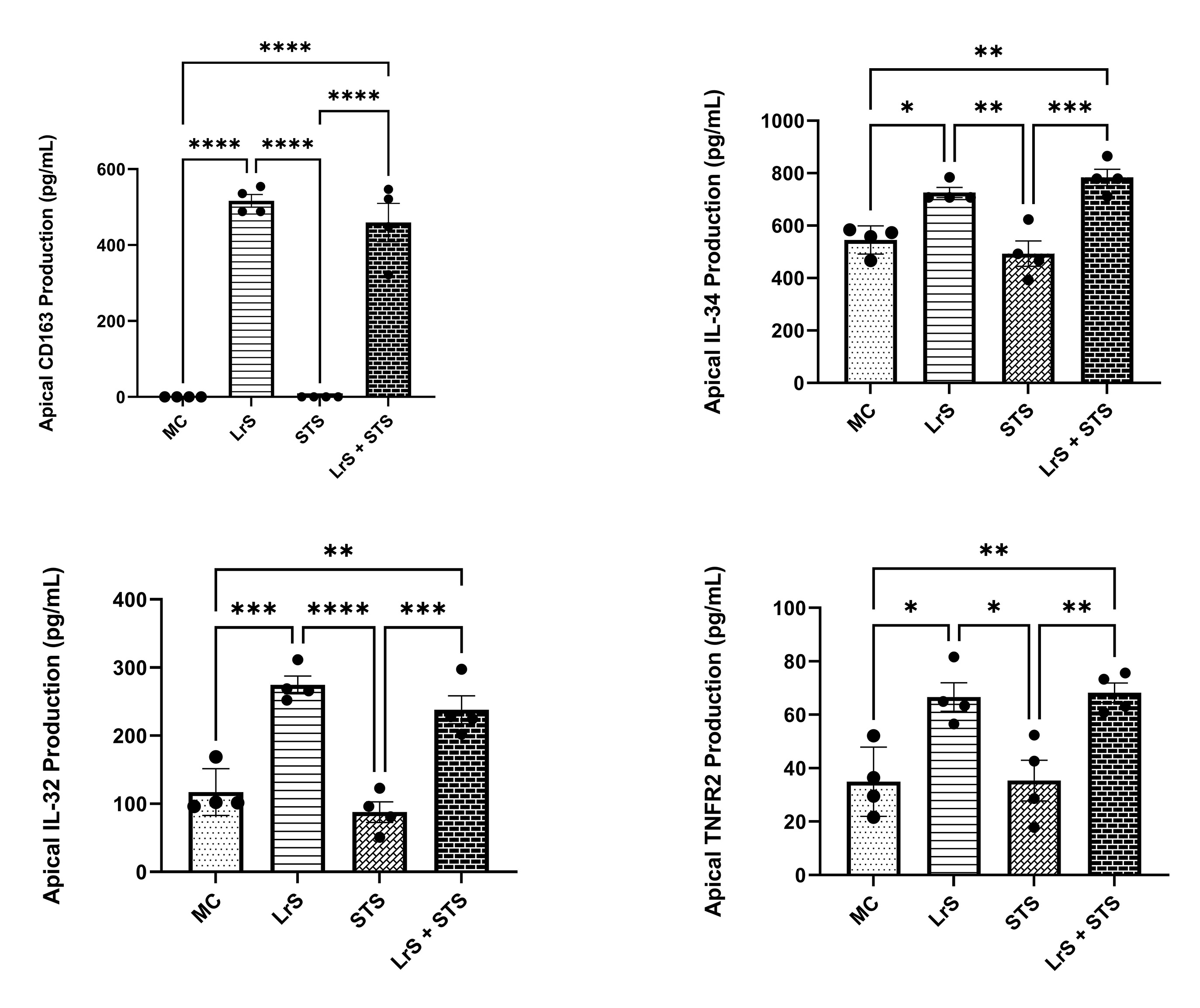

Supplement: Supplementary Figure 3 — Apical production of cytokines and chemokines which were significantly increased by LrS treatment of T84 IEC and THP-1 monocyte co-cultures. Data shown is the mean cytokine/chemokine production (pg/mL) ± SEM (n = 4). Significance is indicated as ∗p < 0.05, ∗∗p < 0.01, ∗∗∗p < 0.001, ∗∗∗∗p < 0.0001 as determined by one-way ANOVA and Tukey’s post-hoc test. [file Image_4.JPEG]
